# Carbon Decorated Pd Species within Zeolite for Selective Furfural Hydrogenation

**DOI:** 10.1021/acscentsci.5c00424

**Published:** 2025-06-23

**Authors:** Risheng Bai, Lin Li, Jingxuan Wang, Shu Miao, Yu Sun, Jihong Yu

**Affiliations:** † State Key Laboratory of Inorganic Synthesis & Preparative Chemistry, College of Chemistry, 12510 Jilin University, Changchun 130012, P. R. China; ‡ School of Advanced Materials and Nanotechnology, Xidian University, Xi’an 710126, P. R. China; § JEOL (Beijing) Co., Ltd., Beijing, 100080, P. R. China; ∥ Electron Microscopy Center, Jilin University, Changchun 130012, P. R. China; ⊥ International Center of Future Science, Jilin University, Changchun 130012, P. R. China

## Abstract

Selective hydrogenation of biomass-derived furfural to furfuryl alcohol is fundamentally challenged by the dual adsorption of its reactive furan ring and carbonyl group on Pd catalysts, which drives nonselective pathways. To modulate the adsorption mode of furfural, we rationally incorporate carbon species onto Pd subnanoclusters encapsulated in a siliceous **MFI** zeolite (Pd-C@S-1), achieving 98% selectivity for furfuryl alcohol at full furfural conversion, vastly outperforming the carbon-free Pd@S-1 (11.6% selectivity). Comprehensive characterizations and theoretical calculations reveal that the enhanced catalytic efficiency of Pd-C@S-1 arises from carbon-induced modifications to the electronic and steric properties of Pd. The resulting Pd-C_3_ species promote a vertical adsorption orientation of furfural via the carbonyl group, sterically hindering interactions between the furan ring and Pd active sites, thus leading to high selectivity for furfuryl alcohol. This work demonstrates that the modification of Pd with carbon species can alter the substrate adsorption configurations, enabling precise control over reaction pathways and offering a facile strategy for designing selective catalysts in biomass conversion.

## Graphical abstract

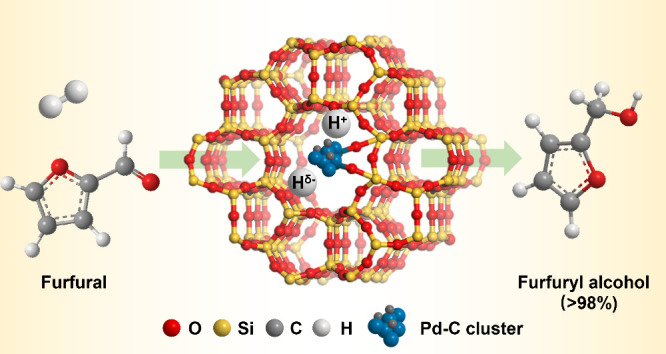

## 1 Introduction

Transforming biomass feedstocks, which are carbon-neutral renewable energy resources, into biofuels and fine chemicals via economically viable processes, plays a critical role in advancing sustainable chemistry.^1–6^ Furfural, obtained from biomass components such as cellulose and hemicellulose, stands as a pivotal platform molecule that connects biomass sources to the industries of biofuels and fine chemicals.^6–14^ An effective strategy for upgrading furfural involves its hydrogenation, such as for producing furfuryl alcohol, tetrahydrofurfuryl alcohol, furan, 2-methyltetrahydrofuran, 2-methylfuran, etc., which hinges upon the functionalities of the heteroaromatic ring and the adjacent aldehyde group.^15–21^ Among the various upgraded products, furfuryl alcohol is predominantly valued for its application in the synthesis of thermally stable and corrosion-resistant furfuryl alcohol resin for the casting industry and as a potential precursor for biobased aviation fuels, underscoring its industrial and sustainable energy significance.^22–24^

Palladium (Pd) and platinum (Pt) are effective in furfural upgrading, yet the adsorption configuration of furfural (e.g., η^1^(C)-acyl, η^1^(O)-aldehyde, and η^2^(C–O)-aldehyde configuration) on these metals critically influences the hydrogenation results.^19,25–28^ For the Pd-based catalyst, the most stable furfural adsorption on Pd (111) surfaces involves the furan ring lying flat and forming strong bonds with the metal. This configuration often results in low selectivity for furfuryl alcohol, as it facilitates undesirable side reactions, including hydrogenolysis, decarbonylation, and ring hydrogenation.^29–33^ Strategies like modulating metal particle sizes and shapes, incorporating metal additives, and tweaking the catalyst surface have proved to be effective for optimizing furfural adsorption and thus enhancing the product selectivity, albeit often at the cost of reducing catalytic efficiency or necessitating harsh catalytic conditions.^34,35^ Tomishige and co-workers introduced a Pd-Ir alloy catalyst that, by modifying furfural adsorption, yields 40.9% furfuryl alcohol at 61.0% conversion under high hydrogen pressure (8 MPa),^36^ underscoring the challenge of finding efficient, mild-condition furfural hydrogenation systems.

Zeolites, characterized by their well-defined microporous structures and exceptional hydrothermal stability, are outstanding for immobilizing metal nanoparticles or even single metal atoms.^37–43^ The zeolite structure successfully prevents the clustering of metal nanoparticles, and its consistent pores regulate the spatial arrangement of reactant molecules, enabling distinctive selectivity in products for a range of reactions.^44–50^ Ion-exchange, wet impregnation, ligand-stabilized, and complex-protected methods are effective in encapsulating metal nanoparticles (such as Pt, Pd, Ru, Rh) into zeolite frameworks.^40,46,51–53^ While narrow zeolite channels and cages can encapsulate nanoclusters and prevent them from sintering under harsh conditions, the narrow space in the zeolite frameworks also limits further electronic and steric modification of the encapsulated metal species.^54^ Thus, developing metal encapsulated zeolite catalysts with controllable metal electronic and steric properties is of significant importance.

In this work, we encapsulate the carbon-decorated subnanometric Pd species within a pure siliceous silicalite-1 (S-1, **MFI**) zeolite matrix. This encapsulation is achieved through a one-pot hydrothermal crystallization method combined with a direct carbonization-reduction protocol, resulting in the Pd-C@S-1 catalyst. The in situ formed carbon species alters the electronic and morphological properties of the embedded metal particles, thereby enhancing their catalytic performance. These improvements, driven by modified adsorption modes of furfural, lead to markedly increased catalytic activity and furfuryl alcohol selectivity during furfural hydrogenation. Remarkably, the carbon-decorated catalysts achieve furfuryl alcohol selectivity of up to 98% at complete furfural conversion, surpassing the performance of their carbon-free counterparts.

## 2 Results and Discussion

Figure 1a summarizes the synthesis protocol for fabricating zeolite-encapsulated carbon-decorated Pd catalysts (Pd-C@S-1) and control samples. The Pd species were first introduced into the siliceous **MFI** zeolite (silicalite-1, S-1) via a one-pot hydrothermal synthesis, leveraging an ethylenediamine complex to ensure uniform dispersion, yielding the precursor Pden@S-1. Subsequent treatment under a hydrogen atmosphere at 390 °C triggered a hydrogen-mediated carbonization-reduction step (Figure S1), facilitating the in situ formation of carbon-modified Pd species (Pd-C) within the zeolite channels. This process culminated in the final Pd-C@S-1 catalyst, where carbon decoration and Pd confinement within the S-1 framework are achieved simultaneously. For the preparation of the control samples without carbon, an initial calcination was first performed in an air atmosphere at 550 °C to eliminate the organic components. This was then followed by reduction under a hydrogen atmosphere at 390 °C. X-ray diffraction (XRD) analysis of all metal-incorporated S-1 zeolite samples reveals characteristic patterns consistent with the **MFI** topology (Figure S2). Remarkably, the diffraction peaks associated with the Pd metals are not detected. This is presumably attributed to their low loading amounts, extremely small particle sizes, and homogeneous distribution. Inductively coupled plasma-optical emission spectroscopy (ICP-OES) determines the Pd loadings of 0.71 and 0.72 wt % for Pd-C@S-1 and Pd@S-1, respectively (Table S1). Scanning electron microscopy (SEM) reveals coffin-like morphologies with particle sizes around 150 nm for both Pd-incorporated samples (Figure S3). High-angle annular dark-field scanning transmission electron microscopy (HAADF-STEM) and conventional TEM analyses elucidated the spatial distribution and dimensions of metal species embedded within the zeolite framework, with carbon-decorated Pd nanoparticles exhibiting an average size of ∼0.9 nm (Figures 1b–e and S4), significantly smaller than that of the carbon-free Pd@S-1 (2.6 nm; Figure S5). High-resolution spherical aberration corrected (Cs-corrected) HAADF-STEM image further uncovers exquisitely dispersed metal clusters consisting of several atoms along with atomically dispersed Pd atoms within Pd-C@S-1 (Figure 1e). Energy-dispersive spectroscopy (EDS) elemental mappings confirmed the uniform distribution of Si, O, and Pd throughout the zeolite crystals (Figure 1f).

**Figure 1:**
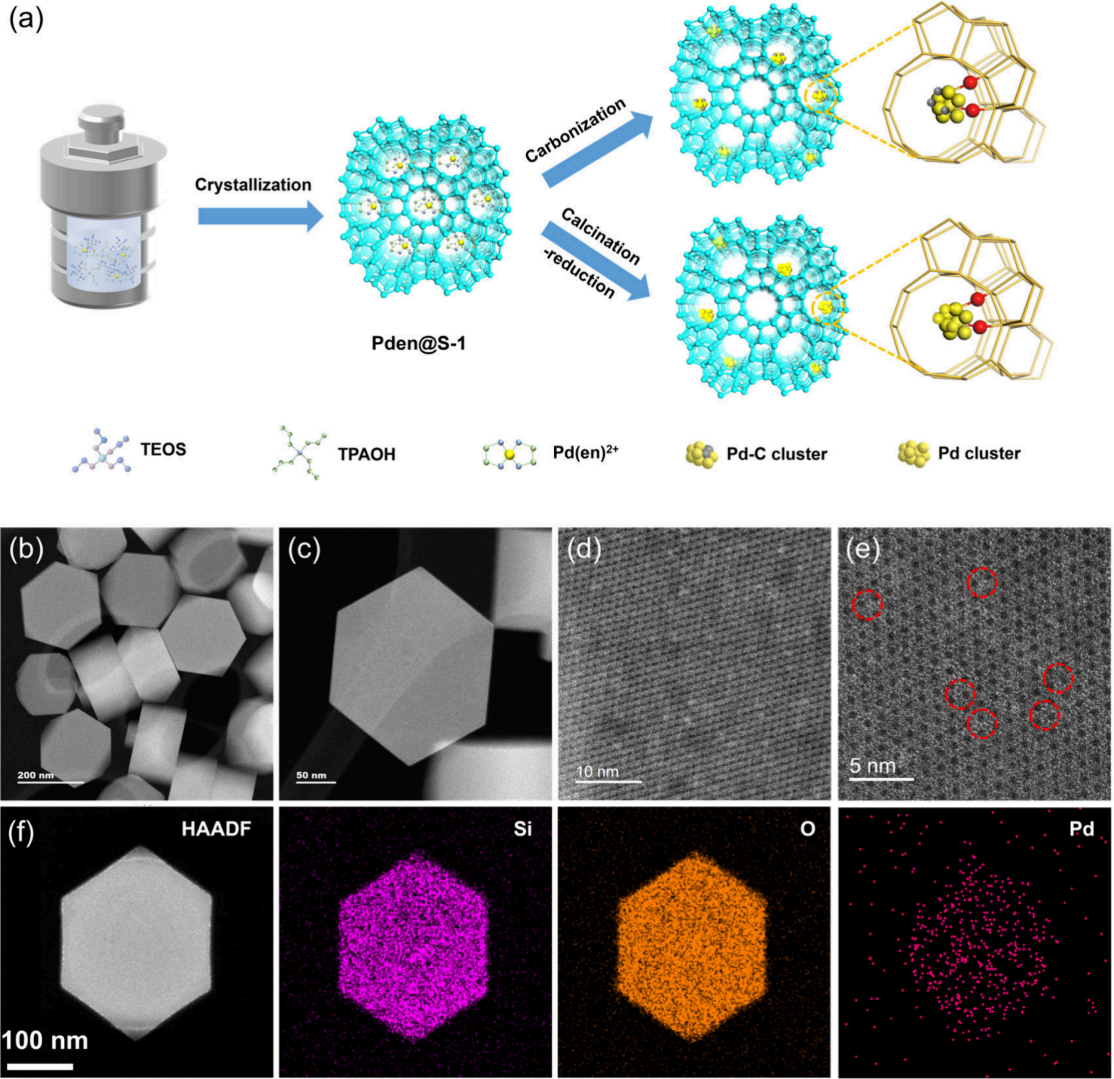
(a) Schematic diagram outlining the synthesis pathway for constructing zeolite-confined Pd-C@S-1. (b–e) Cs-corrected STEM-ADF micrographs highlighting the nanoscale architecture of Pd-C@S-1. (f) HAADF-STEM image paired with elemental distribution maps of Pd-C@S-1.

The chemical states of the encapsulated Pd species within the zeolites were analyzed using in situ diffuse reflectance infrared Fourier transform spectroscopy (DRIFTS) with CO employed as a probe molecule, complemented by X-ray absorption fine structure (XAFS) measurements. The CO-DRIFTS spectra reveal that the peak corresponding to linearly adsorbed CO exhibits higher wavenumbers in the carbon-containing sample (Pd-C@S-1) compared to the carbon-free counterpart (Pd@S-1, Figure 2a). This finding suggests that the Pd species within the carbon-containing sample have a greater positive charge density or may be subjected to dipolar coupling influences. In the CO-DRIFTS spectra of the two Pd-containing samples, the peaks detected at 1950 cm^–1^ and 1953 cm^–1^ are attributed to bridged CO molecules adsorbed on the Pd species, which implies the presence of Pd nanoparticles.

**Figure 2:**
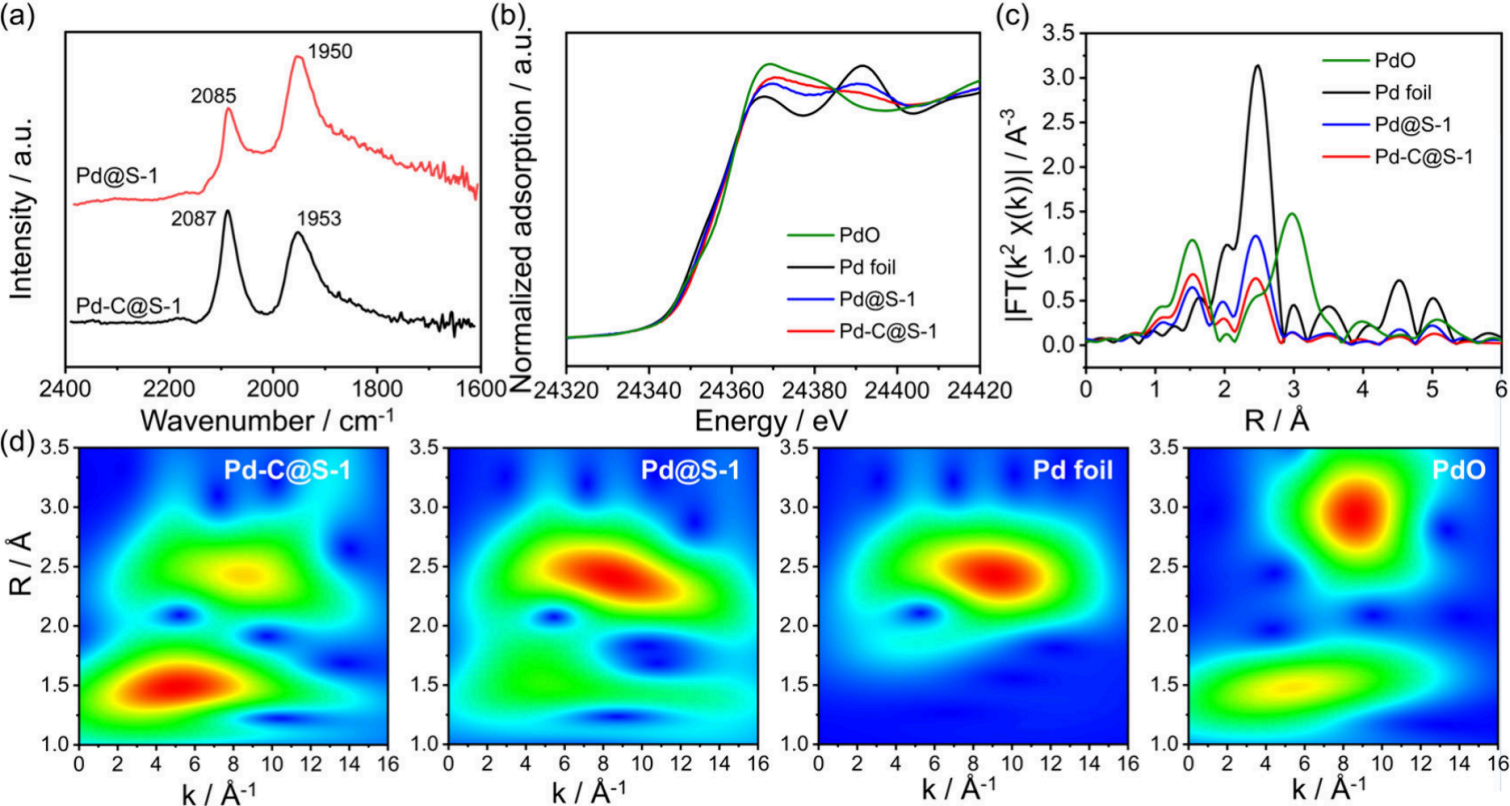
(a) In-situ CO-DRIFTS spectra for encapsulated Pd samples. (b) XANES, (c) FT-EXAFS, and (d) WT-EXAFS of Pd-C@S-1, Pd@S-1, Pd foil, and PdO.

The Pd K-edge XANES spectra for Pd-C@S-1 and Pd@S-1 are shown alongside those of the reference materials in Figure 2b. XANES analysis shows that the white line intensity and absorption edge positions of Pd-C@S-1 and Pd@S-1 lie between those of metallic Pd (Pd foil) and fully oxidized PdO, suggesting a mixed oxidation state for the Pd species in both catalysts. This increased valence state likely results from interaction between Pd and oxygen atoms within the zeolite framework. Notably, Pd-C@S-1 shows a higher white line intensity than Pd@S-1, indicating a more oxidized state of Pd in the carbon-containing sample. The *k* ^2^-weighted extended X-ray absorption fine structure (EXAFS) spectra of Pd-C@S-1 and Pd@S-1 display peaks at 1.53 and 2.46 Å, which are attributed to Pd–C/O and Pd–Pd interactions, respectively (Figure 2c). Wavelet transform (WT) analysis further clarified the EXAFS data by resolving scattering atoms in both the *R*-space and *k*-space (Figure 2d). For Pd-C@S-1 sample, the maximum WT intensity around 4.7 Å^–1^ in *k*-space corresponds to 1.5 Å in *R*-space, indicating Pd-C/O coordination. In contrast, the Pd@S-1 sample shows peak intensity at 8.2 Å^–1^ in *k*-space, corresponding to 2.4 Å in R-space, reflecting dominant metal–metal coordination, consistent with the prevalence of metallic species in carbon-free samples.

Least-squares EXAFS fitting was employed to determine the quantitative coordination environments of Pd species in the synthesized samples. EXAFS fitting results indicate that Pd atoms in Pd-C@S-1 exhibit a coordination number (CN) of ∼2.4, corresponding to bonding with approximately two neighboring Pd atoms at an average distance of 2.73 Å and with three carbon atoms, giving a CN of about 3.1 at a distance of 2.00 Å. These findings suggest the presence of Pd clusters and the formation of Pd-C_3_ complexes (Figure S6 and Table S2). Furthermore, Pd-C@S-1 exhibits a lower coordination number of Pd–Pd shell than the Pd@S-1 sample (2.4 vs 5.4), indicating the smaller Pd species in the former, corroborating the TEM results.

Thermogravimetric analysis (TGA) reveals that Pd-C@S-1 undergoes a weight loss of 0.6 wt % between 300 and 600 °C (Figure S7), confirming the existence of carbon species in the sample, which is further supported by elemental analysis (Table S3). Conversely, the FT-IR spectra do not reveal the presence of organic species, likely due to their low concentrations (Figure S8). The Argon adsorption–desorption isotherms reveal that the carbon-containing sample, Pd-C@S-1, exhibits a slightly reduced volume of Ar adsorption in the relative pressure range of *P*/*P*_0_ < 0.1, when compared to the carbon-free counterpart (Figure 3a), which suggests a decrease in the micropore volume. This trend is further supported by the analysis of cumulative pore volume, pore size distribution, and textural properties, as detailed in Figures 3b and S9 and Table S1. The high crystallinity of Pd-C@S-1, as evidenced by XRD analysis, suggests that the observed reduction in micropore volume within the carbon-containing sample stems predominantly from carbon species obstructing the microporous channels.

**Figure 3:**
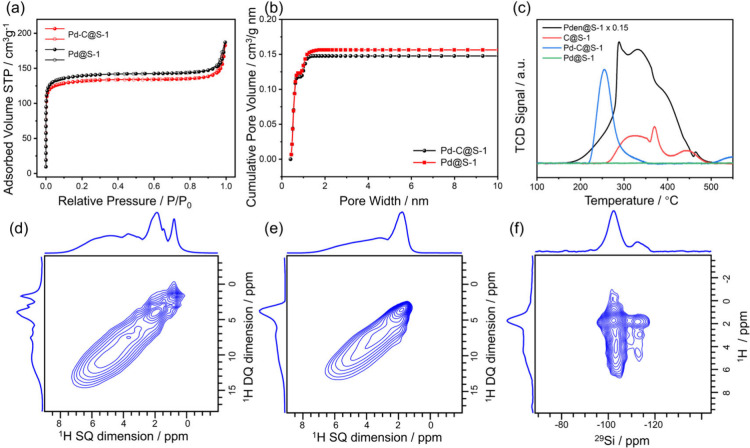
(a) Ar (87 K) isotherms of Pd-C@S-1 and Pd@S-1. (b) Cumulative pore volume derived from the argon (87 K) adsorption isotherms using a DFT model. (c) O_2_-TPO profiles. 2D ^1^H–^1^H DQ MAS NMR spectra of (d) Pd-C@S-1 and (e) Pd@S-1. (f) 2D ^1^H–^29^Si HETCOR spectrum of Pd-C@S-1.

The solid-state ^1^H–^13^C cross-polarization (CP) magic angle spinning (MAS) NMR spectrum of Pd-C@S-1 displays a peak at around 9.0 ppm, which is distinct from the peaks attributed to tetrapropylammonium cations observed in the Pden@S-1 sample (Figure S10).^55^ This difference indicates the formation of novel carbon species during the carbonization process. To gain a more detailed understanding of the organic components, oxygen-temperature-programmed oxidation (O_2_-TPO) analysis was conducted (Figure 3c). In Pden@S-1, oxidation of tetrapropylammonium cations occurs around 290 °C, with additional peaks at 340 and 430 °C. In contrast, Pd-C@S-1 exhibits a single, distinct CO_2_ peak at 255 °C. This differs from the oxidation profile of the carbonized C@S-1 control sample (Figures S11, S12), which shows peaks at 320, 370, and 450 °C. The unique oxidation behavior of Pd-C@S-1 suggests interaction between carbon species and Pd clusters within the zeolite framework.^39^ These findings indicate a structural transformation of organic materials during carbonization in a hydrogen atmosphere, likely due to the catalytic influence of the Pd species.

Solid state NMR measurements over ^1^H and ^29^Si were conducted to investigate the structural characteristics of the zeolite framework. The peaks in ^1^H MAS NMR spectra observed at approximately 1.8, 2.1, and 3.4 ppm are commonly assigned to isolated Si–OH groups, vicinal or geminal Si–OH groups, and hydrogen-bonded silanol groups, respectively (Figure S13). Additionally, Pd-C@S-1 exhibits peaks in the range of 0.8 to 2.2 ppm, confirming the presence of organic species within these samples. The 2D ^1^H–^1^H DQ-SQ MAS NMR spectra (Figure 3d,e) for Pd-C@S-1 and Pd@S-1 display correlation signals in the 3.0–7.0 ppm range, corresponding to silanol nests.^56^ This indicates that the Si–OH structures are preserved across all samples, suggesting that the incorporation of carbon species does not alter the fundamental zeolite framework.

^29^Si MAS NMR spectra of the metal-incorporated samples exhibit signatures at −102 and −112 ppm, correlating with the Q^3^ and Q^4^ silicon atoms, respectively (Figure S14). Notably, the signal at −102 ppm is significantly more prominent in the ^1^H–^29^Si CP MAS NMR spectra for both Pd-C@S-1 and Pd@S-1 samples. This enhancement suggests the presence of Si atom defects in the S-1 crystals, represented as Si­(OH)­(OSi)_3_ (Figure S15). Furthermore, the 2D ^1^H–^29^Si HETCOR spectrum was utilized to reveal the through-space association between ^1^H and ^29^Si nuclei in Pd-C@S-1 (Figure 3f). The spectrum exhibits a strong correlation corresponding to framework Q^3^ Si sites and the H in the neighboring Si–OH groups, underscoring their spatial proximity. Above mentioned observed results imply that the presented carbon species do not interact with the zeolite framework, differing from previously reported instances of Pd carbide encapsulated in S-1, where carbon species decorated both zeolite framework and the encapsulated Pd species.^39^ In Pd-C@S-1, the carbon species predominantly adorn the encapsulated Pd species, which is supported by evidence from CO-DRIFT and XAS analyses and may impact the catalytic performances.

The catalytic performance of Pd-C@S-1 and Pd@S-1 was assessed for furfural hydrogenation under mild conditions (80 °C, 5 bar of H_2_) using H_2_O as the solvent. It is widely recognized that Pd serves as a catalyst for the conversion of furfural into a diverse array of products, as depicted in the reaction pathway outlined in Scheme S1.

With dimensions of 3.371 × 4.556 Å, furfural is capable of penetrating the **MFI** channel pores and undergoing hydrogenation on the surface of the encapsulated Pd species. The Pd-C@S-1 catalyst demonstrates a substantial improvement in performance, achieving a 98% yield of furfuryl alcohol upon complete conversion within a 90 min reaction period. This result significantly surpasses the yields observed for Pd@S-1 (11.9%) and the Pd-free C@S-1 sample (<1%), underscoring the enhanced catalytic efficiency of the Pd-C@S-1 system (Figures 4a and S16). The carbon balances of the catalytic experiments are higher than 93% in each reaction, calculated according to the amount of furan/tetrahydrofuran rings before and after the reactions. After being recycled five times, the Pd-C@S-1 catalyst retained the catalytic activity for furfural and the selectivity of the furfuryl alcohol (Figure 4b). XRD, Ar physisorption, ICP, and TEM measurements suggest that the crystallinity of the zeolite sheath and the Pd species remain almost unchanged (Figures S17–S19 and Table S1). To further elucidate the reaction kinetics, the initial rates of furfural hydrogenation were analyzed at various temperatures. Arrhenius plots (Figure 4c) constructed from these data reveal apparent activation energies of 42.4 kJ mol^–1^ for Pd-C@S-1 and 78.5 kJ mol^–1^ for Pd@S-1. Additionally, the Eyring–Polanyi analysis (Figure 4d) indicates activation enthalpies of 39.3 kJ mol^–1^ for Pd-C@S-1 and 75.5 kJ mol^–1^ for Pd@S-1. The entropy decrease of furfural is significantly greater for Pd-C@S-1 (−105.1 J mol^–1^K^–1^) compared to Pd@S-1 (−25.0 J mol^–1^K^–1^), suggesting that Pd-C@S-1 facilitates furfural hydrogenation more effectively due to a more favorable entropic environment.

**Figure 4:**
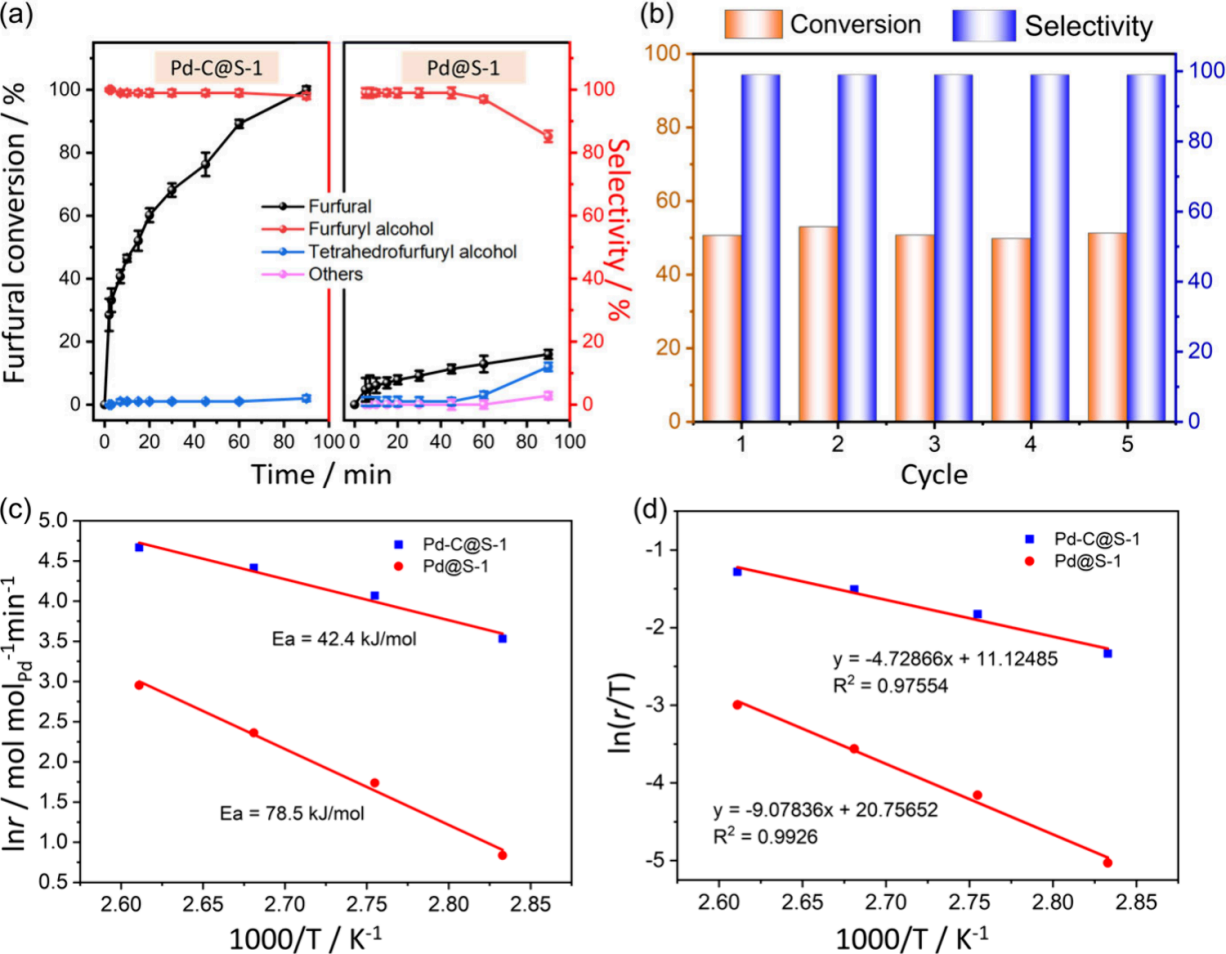
(a) Furfural conversion and furfuryl alcohol selectivity over Pd-C@S-1 and Pd@S-1. Reaction conditions: 0.61 mmol of furfural, 30 mg of catalyst, 10 mL of H_2_O, 5 bar of H_2_, 80 °C. (b) Stability test over Pd-C@S-1. (c) Arrhenius plots and (d) linearized Eyring–Polanyi plot of the furfural hydrogenation over Pd-C@S-1 and Pd@S-1.

To gain deeper insights into the interaction between furfural and the catalysts, we conducted furfural temperature-programmed desorption (TPD) analysis over the two Pd-encapsulated samples (Figure S20). The furfural on Pd-C@S-1 exhibits lower desorption temperatures than the C-free sample (150 and 225 °C vs 190 and 230 °C). Additionally, the total furfural adsorption capacity of Pd-C@S-1 is significantly reduced in comparison to Pd@S-1. These findings imply that Pd-C@S-1 demonstrates a lower avidity toward furfural. It is postulated that this phenomenon can be ascribed to perturbations in the electronic characteristics of the Pd species, which are instigated by the incorporation of carbon.

To further elucidate the interaction between the Pd species and H_2_, we performed H_2_-DRIFT and H_2_-TPD analyses. The H_2_-DRIFT spectrum of Pd-C@S-1 reveals a sequence of IR bands within the wavenumber range of 2970 to 3900 cm^–1^, which are associated with the stretching vibrations of hydroxyl groups. Additionally, bands in the range of 1900 to 2350 cm^–1^ are discerned, and these are ascribed to the Pd-H species. (Figure S21). These observations strongly suggest the heterolytic dissociation of H_2_ over Pd-C@S-1. In contrast, Pd@S-1 exhibits prominent signals from silanol groups and a weaker Pd-H band, indicating that hydrogen activation on Pd species predominantly forms surface hydroxyls through hydrogen spillover. H_2_-TPD analysis further reveals that Pd-C@S-1 displays a higher H_2_ desorption temperature and increased hydrogen uptake relative to Pd@S-1 (Figure S22). These results indicate that Pd species with a positive charge density in Pd-C@S-1 are more effective at activating H_2_ molecules, enhancing hydrogenation performance. This superior activity can be attributed to the electronic effects introduced by carbon incorporation during the carbonization process.

The exceptional catalytic performance of selective furfural hydrogenation over carbon-decorated Pd encapsulated in pure silicate **MFI** is further elucidated through first-principles density functional theory (DFT) calculations (Figure 5 and Figures S23, S24). This study compares the furfural hydrogenation over Pd-C@S-1, which contains carbon-decorated Pd nanoclusters, and Pd@S-1, which features Pd nanoparticles. Bader charge analysis and charge density difference calculations over Pd-C@S-1 and Pd@S-1 zeolite samples are demonstrated in Figure 5a,b. The Pd species in Pd-C@S-1 exhibits a positive charge of +0.64 |e|, whereas Pd in Pd@S-1 carries a lower positive charge of +0.05 |e|, confirming significant electron transfer from Pd to adjacent carbon atoms in Pd-C@S-1, resulting in an electron-deficient Pd state. The charge density difference map (Figure 5a,b) visualizes electron depletion (blue) at Pd sites and electron accumulation (yellow) around neighboring carbon atoms, directly corroborating the Bader charge results. The results are in line with the CO-DRIFT spectra and XAS characterizations. The reaction energy profiles and corresponding structural configurations are depicted in Figures 5c, S25, and S26. Notably, the furfural molecule adsorbs in a stable η^1^-(O)-aldehyde configuration on the Pd-C@S-1 catalyst with an adsorption energy of −0.42 eV, where the oxygen of the carbonyl group interacts with the Pd-C species. However, in the case of Pd@S-1, furfural adopts a stable η^2^-(C)-acyl configuration with an adsorption energy of −0.31 eV, where the furan ring lies flat on the Pd nanoparticle surface. This difference suggests that the aldehyde functionality on Pd-C@S-1 is more readily reactive with adsorbed hydrogen atoms, whereas this adsorption mode limits the conversion of the furan ring. The tilted adsorption mode in Pd-C@S-1 arises from two synergistic factors: (1) electronic modulation: carbon incorporation induces a higher oxidation state of Pd (+0.64 |e| vs +0.05 |e|, Bader charge analysis), as corroborated by CO-DRIFT and XAS data, which strengthens Pd–O interactions; (2) confinement effects: The carbon-modified microporous zeolite framework (evidenced by TGA and Ar physisorption) restricts reactant mobility, stabilizing the tilted orientation. Upon dissociation of the adsorbed H_2_, the Pd-C@S-1 catalyst achieves an electronic energy change of −2.10 eV, significantly lower than that of Pd@S-1 (−1.83 eV), indicating that Pd-C@S-1 facilitates the dissociation of H_2_ molecules more efficiently. This is consistent with the H_2_-TPD results and previous reports.^39^ Additionally, the reaction barriers for hydrogenating the aldehyde group over Pd-C@S-1 and Pd@S-1 are 0.74 and 0.85 eV, respectively, further confirming that Pd-C@S-1 is more efficient in catalyzing this hydrogenation step, in alignment with experimental findings. It is also important to highlight that the energy barriers for the additional hydrogenation of furfuryl alcohol are 1.00 eV for Pd-C@S-1 and 1.10 eV for Pd@S-1, suggesting that further hydrogenation is less effective on Pd-C@S-1 compared to Pd@S-1. This accounts for the higher selectivity of Pd-C@S-1 toward the furfuryl alcohol. Further, we emphasize that catalytic performance is influenced not only by hydrogenation energetics but also by solvent/reactant adsorption and product/intermediate desorption—factors not fully captured in our current DFT framework. These complexities likely contribute to the high furfuryl alcohol selectivity observed experimentally. Our findings collectively highlight the necessity of holistic catalyst design that integrates electronic modulation (as evidenced by DFT) with microenvironmental effects (e.g., adsorption/desorption dynamics) to optimize catalytic behavior.^57–59^

**Figure 5:**
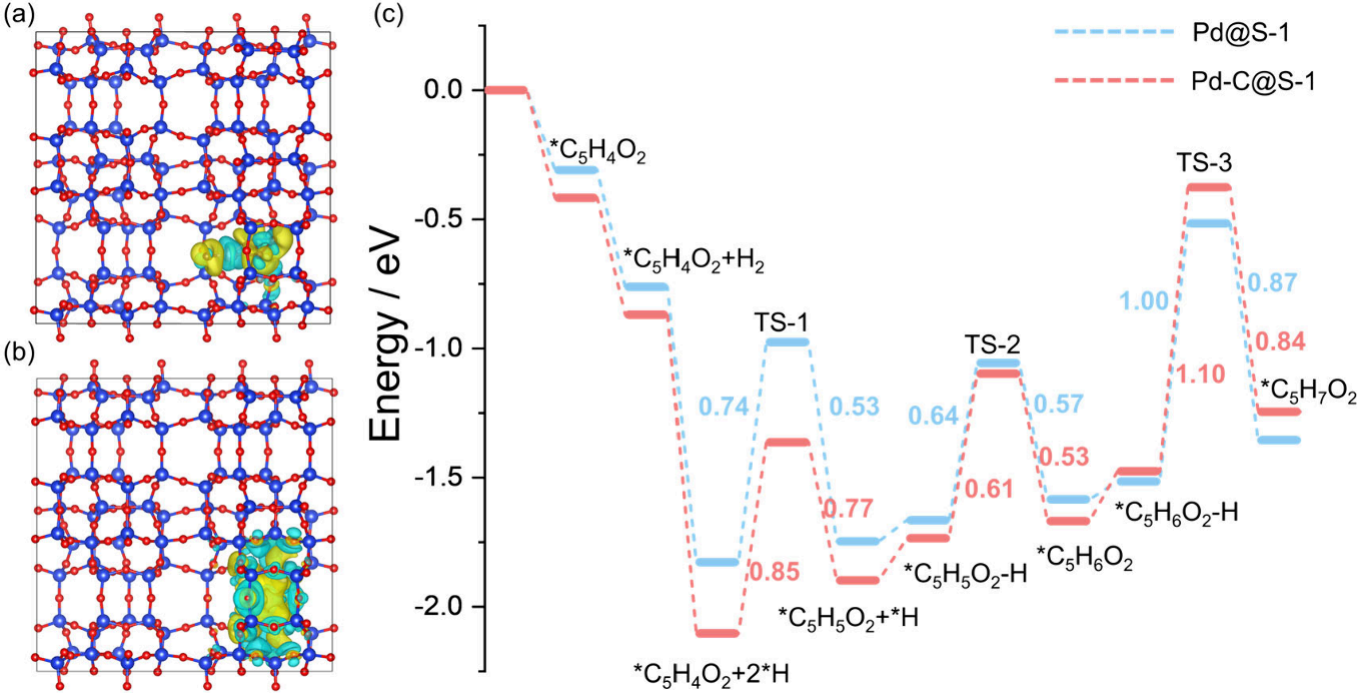
Differential charge densities of (a) Pd-C@S-1 and (b) Pd@S-1. (c) DFT calculated reaction energy profiles for selective hydrogenation of furfural over Pd-containing samples.

## 3 Conclusions

In summary, we have developed a straightforward carbonization-reduction approach to encapsulating carbon-decorated subnanometric Pd species within purely siliceous **MFI** zeolites. Comprehensive analyses by means of NMR, XAS, and CO-DRIFTS techniques validate the generation of Pd-C structures and uncover the existence of positively charged Pd species. The carbon-decorated catalyst affords up to 98% selectivity for furfuryl alcohol at full furfural conversion, significantly outperforming the carbon-free counterpart. The superior catalytic performance can be ascribed to the optimized furfural adsorption mode and the enhanced H_2_ activation over Pd active sites, as confirmed by detailed characterizations and theoretical calculations. These findings provide valuable insights in the development of high-performance catalysts for selective hydrogenation reactions.

## Supplementary Material



## Notes

Experimental procedure details and methods, XRD patterns, SEM images, TEM images, EXAFS fitting curves, and ^1^H MAS NMR spectra of the prepared samples (PDF)
