# Supplementary material for: Carbon Decorated Pd Species within Zeolite for Selective Furfural Hydrogenation
Source: ACS Cent Sci. 2025 Jun 23;11(8):1336–44. doi: 10.1021/acscentsci.5c00424 (PMC12395294; doi:10.1021/acscentsci.5c00424)
Supplement: Supplementary file 1 [file oc5c00424_si_001.pdf]

Supporting Information

Carbon Decorated Pd Species within Zeolite for Selective Furfural Hydrogenation

*Risheng Bai, Lin Li, Jingxuan Wang, Shu Miao, Yu Sun, and Jihong Yu**

*Correspondence:
jihong@jlu.edu.cn (J. Yu)

Table of Contents

Experimental section... S2
Characterizations.. S4
Catalytic tests.. S8
Computational details S9
Supplementary figures and tables S11
References... S41

Experimental section

Chemical reagents.

All chemicals and materials were obtained from commercial sources and used without further purification unless otherwise specified. Tetrapropylammonium hydroxide solution (TPAOH, 40 wt%) was purchased from Shanghai Adamas Reagent Co. Tetraethylorthosilicate (TEOS) and ethylenediamine ($NH_2CH_2CH_2NH_2$) were purchased from Sinopharm Chemical Reagent Co. Palladium chloride ($PdCl_2$) was purchased from Shanghai Chemical Reagent Co. Furfural (99%) and furfuryl alcohol (98%) were obtained from Aladdin Chemical Co. Deionized (DI) water was from Milli-Q integral water purification system (Millipore, 18.2 $M\Omega \cdot cm^{-1}$).

Preparation of $[Pd(NH_2CH_2CH_2NH_2)_2]Cl_2$ solution.

The solution of $[Pd(NH_2CH_2CH_2NH_2)_2]Cl_2$ was prepared by mixing 0.322 g of $PdCl_2$ with 8.0 g of H_2O and 2 mL of ethylenediamine under ultrasonic at room temperature for complete dissolution.

Synthesis of Pden@S-1 sample.

The Pden@S-1 sample was prepared through a one-pot synthesis method with the gel composition of SiO_2: 0.3 TPAOH: 4.5×10^{-3} $[Pd(NH_2CH_2CH_2NH_2)_2]Cl_2$: 28 H_2O under hydrothermal conditions at 170 °C for 48 h. Typically, a TPAOH solution was prepared by mixing 3.048 g of TPAOH and 8.971 g of distilled water at room temperature. Then, 0.42 mL of $[Pd(NH_2CH_2CH_2NH_2)_2]Cl_2$ solution was added into the TPAOH solution and stirred for 10 min at room temperature. After that, 4.167 g tetraethylorthosilicate (TEOS) was added into the mixture and stirred for 12 h at room temperature to get the full hydrolysis (1000 rpm). The resultant solution was then transferred into Teflon-lined autoclaves and crystallized at a preheated oven of 170 °C for 48 h under static conditions. After the hydrothermal crystallization process, the solid product was isolated by centrifugation, thoroughly washed with distilled water and ethanol and then dried at 80 °C.

Synthesis of Pd-C@S-1.

The Pd-C@S-1 sample was prepared by direct carbonization and reduction of the obtained Pden@S-1 sample at 390 °C for 2 h under an H_2 atmosphere with a heating rate of 3 °C/min and a gas flow rate of 40 mL/min.

Synthesis of Pd@S-1.

The Pd@S-1 sample was prepared by calcining the obtained Pden@S-1 sample in air at 550 °C for 6 h (with a heating rate of 1.5 °C/min), followed by reduction at 390 °C for 2 h under H_2 atmosphere with a heating rate of 3 °C/min and a gas flow rate of 40 mL/min.

Synthesis of C@S-1.

The S-1 sample was prepared through a one-pot synthesis method with the gel composition of SiO_2: 0.3 TPAOH: 1.08×10^{-2} $NH_2CH_2CH_2NH_2$: 28 H_2O under hydrothermal conditions at 170 °C for 48 h. The C@S-1 sample was prepared by direct carbonization of the obtained S-1 sample at 390 °C for 2 h under an H_2 atmosphere with a heating rate of 3 °C/min and a gas flow rate of 40 mL/min.

Characterizations

X-ray diffraction (XRD)

Powder X-ray diffraction analysis of the samples was carried out on a Rigaku D-Max 2550 diffractometer using Cu Kα radiation (λ = 1.5418 Å, 50 KV).

Scanning electron microscopy (SEM)

Scanning electron microscopy (SEM) experiments were performed with JEOL JSM-6700F.

Transmission electron microscopy (TEM)

Transmission electron microscopy (TEM) images were recorded on JEM-2100F and Tecnai F20 electron microscope.

Spherical aberration-corrected scanning transmission electron microscopy (Cs-corrected STEM)

Spherical aberration-corrected (Cs-corrected) scanning transmission electron microscopy (STEM) data were acquired on a JEOL GrandARM 300 instrument equipped with a double corrector. The microscope was equipped with a field-emission gun (FEG), two JEOL correctors, a JEOL EDS, and a Gatan quantum energy filter for spectroscopic analyses. Prior to observation, the STEM corrector was aligned using a thin amorphous carbon layer, assuring a spatial resolution of 0.7 Å. Sample preparation consisted of embedding the zeolite granules in resin and trimming with a microtome (Ultracut) prior to Cs-corrected HRTEM measurement.

The average Pd particle size is calculated according to $d = \Sigma nidi^3/\Sigma nidi^2$, where ni stands for the number of the particle with a size of di.

Argon physisorption

Argon adsorption-desorption measurements were carried out on a Micromeritics ASAP 3-flex analyzer at 87 K. Before starting the Ar adsorption measurements, all the samples were activated by degassing in-situ at about 473 K for 10 h.

Inductively coupled plasma (ICP)

Chemical compositions were determined with Inductively Coupled Plasma-Optical

Emission Spectrometry (ICP-OES) analysis performed on an iCAP 7000 Series.

Fourier transform infrared spectra (FT-IR)

Fourier Transform infrared spectra (FT-IR) were recorded by Bruker vertex 80v Infrared Instrument using KBr pellet technique.

***in situ* diffuse reflectance infrared Fourier transformed spectra (*in situ*-DRIFTS)**

In situ diffuse reflectance infrared Fourier transformed spectra (DRIFTS) experiments were performed on a Nicolet iS50 FT-IR spectrometer equipped with a highS4 temperature DRIFTS reaction cell (Harrick Scientific Products, INC) using an MCT detector in the series mode with 32 scans and at a resolution of 4 cm^{-1}. Typically, in situ DRIFTS of CO adsorption experiments, 50 mg catalyst was first activated in pure H_2 flow (15 mL/min) at 673 K (with linear heating to 673 K for 1 h and holding for 1 h). Following, the gas flow was switched to pure N_2 (15 mL/min) for 1 h until the system was cooled down to room temperature. After taking the background spectrum under N_2 atmosphere at room temperature, 5% CO balance with Ar (15 mL/min) was admitted for 1 h until saturated. After CO was fully adsorbed on the metal-containing samples, nitrogen was introduced to remove the physically adsorbed CO, and the peaks for linear-type and bridging-type CO gradually appeared.

In situ DRIFTS of H_2 adsorption experiments, 50 mg catalyst was first activated in pure H_2 flow (15 mL/min) at 473 K (with linear heating to 473 K for 1 h and holding for 1 h). Following, the gas flow was switched to pure N_2 (15 mL/min) for 1 h until the system was cooled down to room temperature. After taking the background spectrum under N_2 atmosphere at room temperature, 10% H_2 balance with Ar (15 mL/min) was admitted.

Thermogravimetric Analysis (TGA)

Thermogravimetric analysis (TGA) was performed with a TA company TGA Q500 in air atmosphere with a heating rate of 10 °C/min from room temperature to 800 °C.

Temperature-programmed desorption (TPD)

The experiment of temperature-programmed desorption of furfural (furfural-TPD) and dihydrogen (H_2-TPD) were measured on a Micromeritics AutoChem II 2920 instrument. For the furfural-TPD measurements, typically, 100 mg of samples were used for each

measurement. The sample was pretreated at 390 °C in an H_2 gas flow for 60 min and then with He gas flow for 30 min. The sample was then cooled to 50 °C and furfural pulses were injected from a calibrated on-line sampling valve. The weakly adsorbed furfural were subsequently removed by purge with He for 60 min. Subsequently, the temperature was raised to 300 °C at a rate of 10 °C/min in the flowing He and the furfural-TPD profile was recorded by the TCD detector.

Temperature-programmed oxidation (TPO)

The temperature-programmed oxidation experiment was measured on a Micromeritics AutoChem II 2920 instrument. Typically, 100 mg of samples were used for each measurement. The sample was pretreated at 390 °C in an He gas flow for 60 min and then cooled to at 50 °C. Subsequently, the temperature was raised to 600 °C at a rate of 10 °C/min in the flowing O_2 and the O_2-TPD profile was recorded the TCD detector.

Solid-state magic angle spinning nuclear magnetic resonance (MAS-NMR)

The ^{1}H, ^{13}C, and ^{29}Si solid-state MAS NMR experiments were performed at 14.09 T on a Bruker Avance Neo 600WB spectrometer at resonance frequencies of 600.23, 150.94, and 119.24 MHz, respectively, with a magic angle spinning rate of 10 kHz. Single-pulse ^{1}H MAS NMR experiments were performed using a π/2 ^{1}H pulse length of 4.0 μs, a repetition time of 2 s, and 32 scans. For the two-dimensional (2D) ^{1}H-^{1}H double quantum (DQ) single quantum (SQ) MAS NMR measurements, DQ coherences were excited and reconverted with a POST-C7 pulse sequence.[1] The increment interval in the indirect dimension was 80 μs. Typically, 128 scans were acquired for each t_1 increment, and two-dimensional data sets consisted of 128 t_1 × 256 t_2. The ^{1}H MAS NMR signals were referenced to adamantane (1.82 ppm).

Single-pulse ^{29}Si MAS NMR spectra with high power proton decoupling were recorded on a 3.2 mm probe, using a π/2 pulse of 5.1 μs, a recycle delay of 80 s and 128 scans.

^{1}H-^{29}Si CP MAS spectra and two-dimensional ^{1}H-^{29}Si HETCOR NMR spectra were acquired with a recycle delay of 2 s and a contact time of to 4 ms.[2-4] The ^{29}Si MAS NMR spectra were referenced to kaolinite (-91.5 ppm).

^{1}H-^{13}C CP MAS spectra were recorded on a 3.2 mm probe, with a recycle delay of 2 s and

contact time of 2.5 ms. The ^{13}C MAS NMR spectra were referenced to adamantane (38.5 ppm).

X-ray absorption spectroscopy (XAS)

The data of X-ray absorption near-edge structure (XANES) and extended X-ray absorption fine structure (EXAFS) were collected at room temperature in the fluorescent mode with a Lytle detector at beamline BL14W1 of the Shanghai Synchrotron Radiation Facility (SSRF, China). The samples were measured in fluorescence mode, using a Lytle detector to collect the data. Data processing and EXAFS fitting were performed using the Athena, Artemis and Igor software.

Catalytic tests

Furfural hydrogenation

The hydrogenation of furfural (FFL) was performed in a high-pressure autoclave equipped with a magnetic stirrer. In a typical procedure, 30 mg of catalyst, 0.61 mmol of FFL and 10 mL of deionized water were well mixed in the autoclave. The reaction system was first purged with pure H_2 ten times, then pressured to 0.5 MPa and followed by heating to desired temperature at a stirring rate of 1000 rpm to eliminate the external mass transfer limitation. After the reaction, the product was taken out and extracted several times followed by analyzing by gas chromatography (Agilent 7890B GC System) equipped with HP-innowax column (30 m × 320 μm × 0.25 μm) and FID detector. n-Dodecane was used as an internal standard for quantification. The conversion, selectivity and reaction rate are defined as follows:

$$\text{Conversion (\%)}=\frac{\text{(initial moles -final moles)}}{\text{(initial moles)}}\times 100\%$$

$$\text{Selectivity (\%)}=\frac{\text{(moles of the product)}}{\text{(moles of the all products)}}\times 100\%$$

Mass balances were accurate to within 5%.

The activation energy Ea, is calculated from the Arrhenius equation:

$$k = A\mathrm{e}^{-Ea/RT} \qquad (1)$$

Where: k is the observed rate constant; A is the pre-exponential factor; Ea is the activation energy (J mol^{-1}); R is the universal gas constant (8.314 J $\text{mol}^{-1}\text{K}^{-1}$); and T is the temperature (K).

The enthalpy, $\Delta H^{\neq}$, and the entropy, $\Delta S^{\neq}$, can be calculated from the Eyring-Polanyi equation:

$$k = \frac{k_B T}{h} e^{-\frac{\Delta G^{\neq}}{RT}} \qquad (2)$$

where: k is the observed rate constant; k_B is the Boltzmann constant (1.381×10^{-23} J K^{-1}); T is the temperature (K); h is the Planck's constant (6.626×10^{-34} J s); $\Delta G^{\neq}$ is the Gibbs energy of activation (J mol^{-1}); R is the universal gas constant (8.314 J mol^{-1} K^{-1}); and T is the temperature (K).

The Gibbs energy of activation during a reaction is equal to the change in the enthalpy minus the product of the temperature and the change in the entropy of the system.

$$\Delta G^{\neq} = \Delta H^{\neq} - T\Delta S^{\neq} \qquad (3)$$

Therefore, the Eyring-Polanyi equation can be linearized as follows:

$$\ln\left(\frac{k}{T}\right) = \frac{-\Delta H^{\neq}}{R}\left(\frac{1}{T}\right) + \ln\left(\frac{k_B}{h}\right) + \frac{\Delta S^{\neq}}{R} \qquad (4)$$

Thus, a plot of ln(*k*/*T*) versus $1/T$ produces a straight line with gradient equal to $\frac{-\Delta H^{\neq}}{R}$ and with a y-intercept of $\ln\left(\frac{k_B}{h}\right) + \frac{\Delta S^{\neq}}{R}$.

Stability test

The hydrogenation reaction was performed under the same reaction conditions as described above, except that the recycled catalyst was used. After reaction, the catalyst was washed 10 times with ethanol (20 mL per wash) followed by 3 times with chloroform (20 mL per wash) to ensure complete removal of adsorbed reactants/products. Then, the recycled catalyst was dried under vacuum at 373 K for 12 hours to ensure solvent removal and restore its active surface, weighed and reused in the next experiment run.

Computational details.

First-principles calculations were carried out on the basis of periodic DFT using a generalized gradient approximation within the Perdew-Burke-Ernzerhof exchange correction functional by VASP. The wave functions were constructed from the expansion of plane waves with an energy cutoff of 450 eV. Gamma centered k-piont of 2 × 2 × 3 have been used for geometry optimization. The consistence tolerances for the geometry optimization are set as 1.0 $\times 10^{-5}$ eV/atom for total energy and 0.05 eV/Å for force, respectively. In free energies calculations, the entropic corrections and zero-point energy (ZPE) have been included. The free energy of species was calculated according to the standard formula:

$$\Delta G = E + \Delta ZPE + \Delta H - \Delta TS$$

where ZPE was the zero-point energy, ΔH was the integrated heat capacity, T was the temperature of product, and S was the entropy. Transition state searching was simulated through chain-of-states search method by climbing image nudge elastic band (CI-NEB) script. The convergence of CI-NEB was set to 0.05 eV/Å for force with 8 intermediate images.

Supplementary Figures and Tables

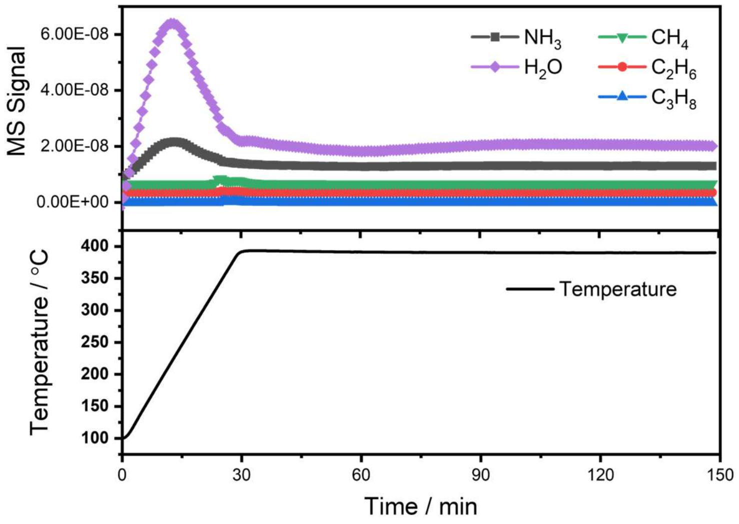

Figure S1. The relationship between temperature and the treatment time during the H_2 carbonization-treatment process and the MS signals for Pden@S-1.

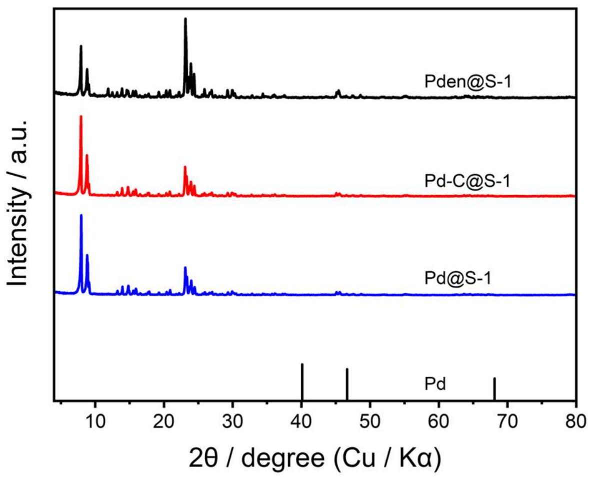

Figure S2. XRD patterns of Pd-containing samples.

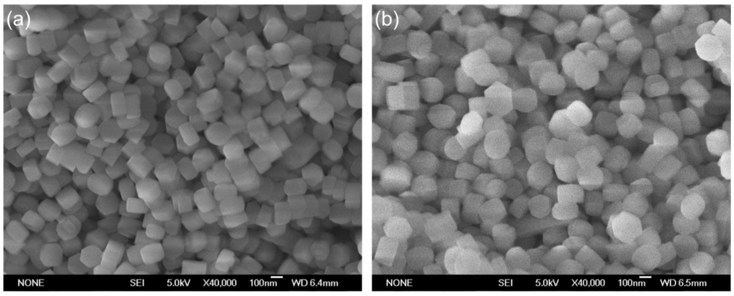

Figure S3. SEM images of (a) Pd-C@S-1 and (b) Pd@S-1.

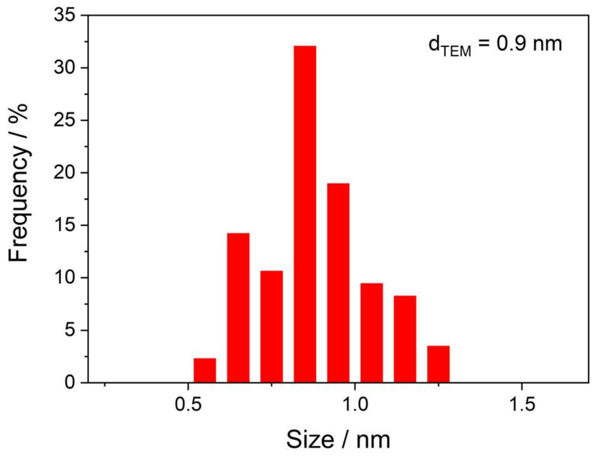

Figure S4. Size distribution of Pd species in Pd-C@S-1.

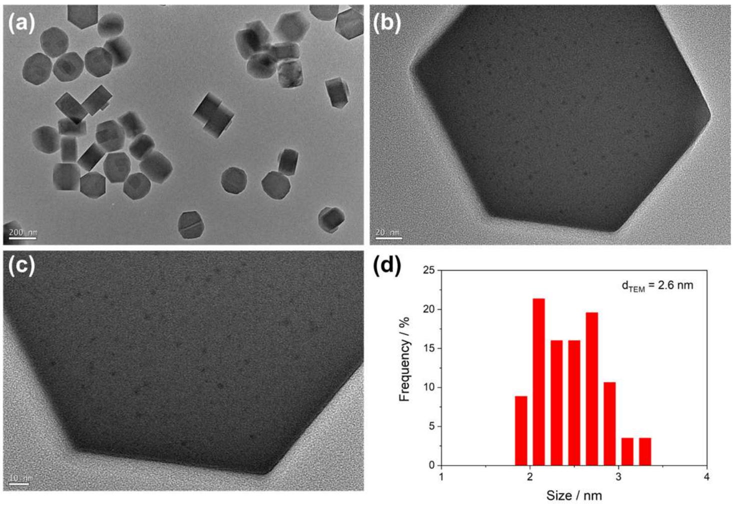

Figure S5. (a-c) TEM images of Pd@S-1, and (d) the size distribution of Pd species in Pd@S-1.

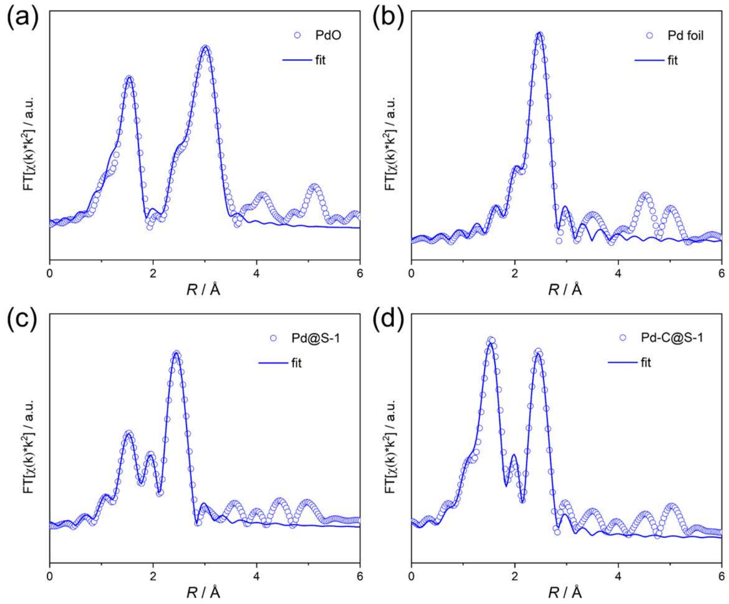

Figure S6. EXAFS fitting curves of Pd K-edge for (a) PdO, (b) Pd foil, (c) Pd@S-1, and (d) Pd-C@S-1 in R space.

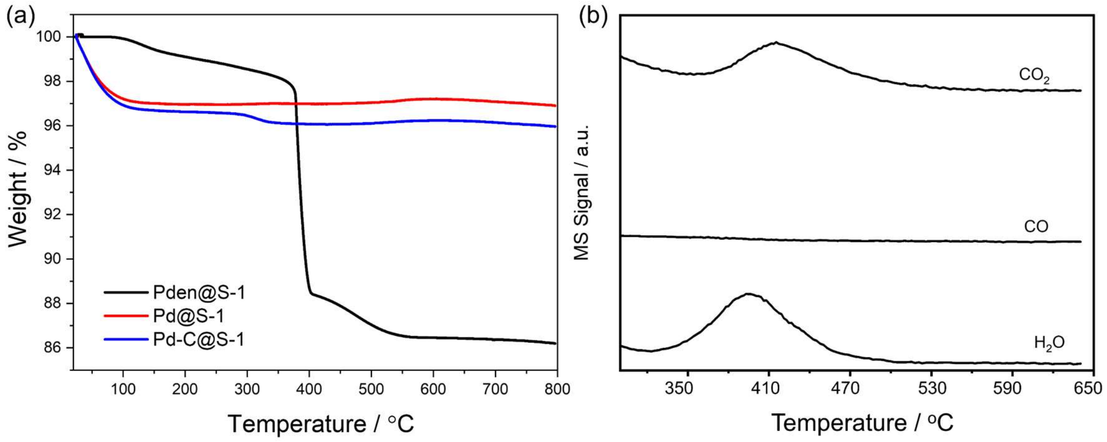

Figure S7. (a) TG curves of the Pden@S-1, Pd-C@S-1, and Pd@S-1 samples. (b) MS signals of Pd-C@S-1 sample.

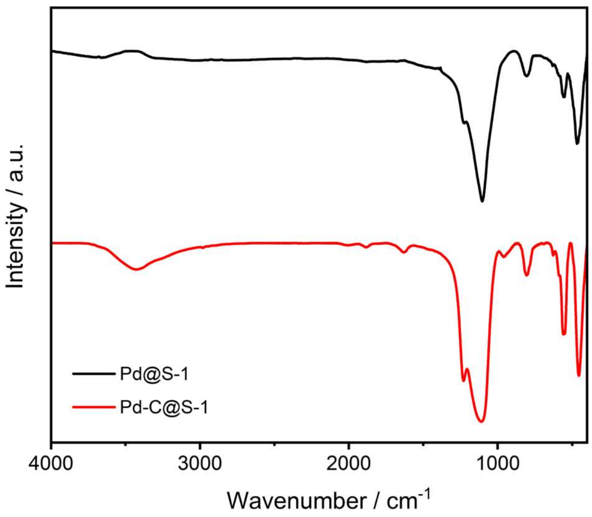

Figure S8. FT-IR spectra of Pd@S-1 and Pd-C@S-1.

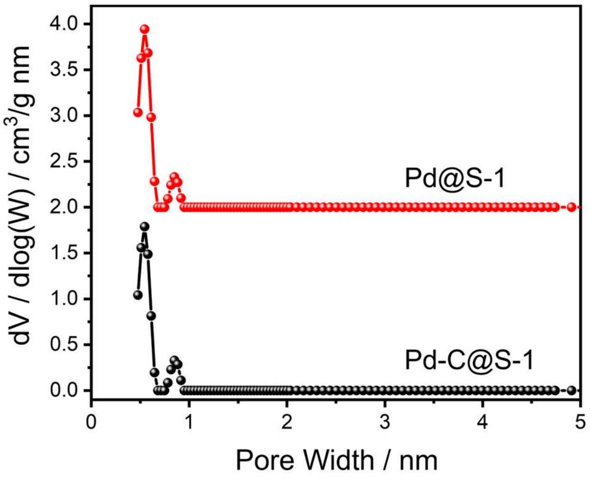

Figure S9. Pore size distribution of Pd-C@S-1 and Pd@S-1.

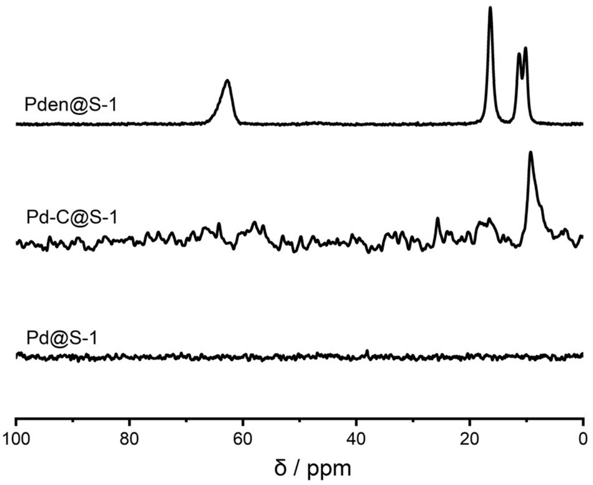

Figure S10. Solid-state ^{1}H-^{13}C CP MAS NMR spectra of the Pd-encapsulated samples.

Note: ^{13}C CP MAS NMR spectrum of Pden@S-1 sample shows the peaks at 10.2 and 11.3 ppm, 16.4 ppm, and 62.9 ppm, which are ascribed to the C_{γ}, C_{β}, and C_{α} in TPA^{+} molecules, respectively.

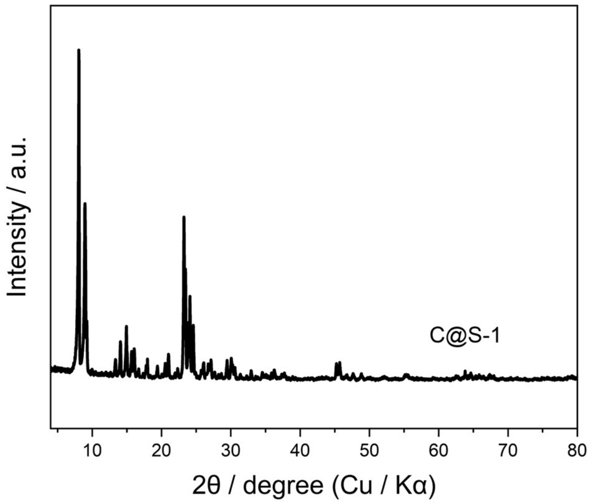

Figure S11. XRD pattern of the prepared C@S-1 sample.

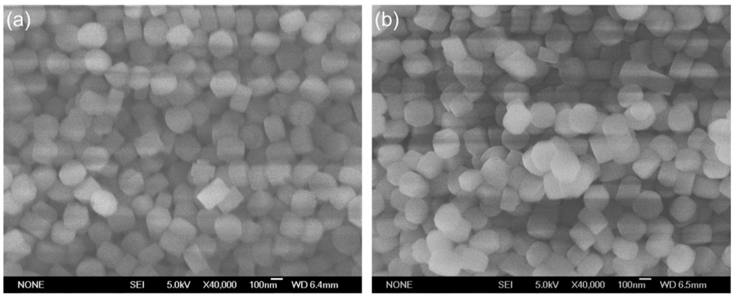

Figure S12. SEM images of the prepared C@S-1 sample.

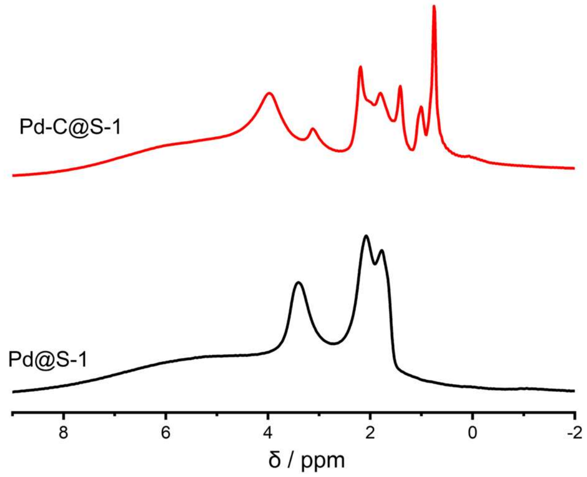

Figure S13. ^1H MAS NMR spectra of Pd-C@S-1 and Pd@S-1 samples.

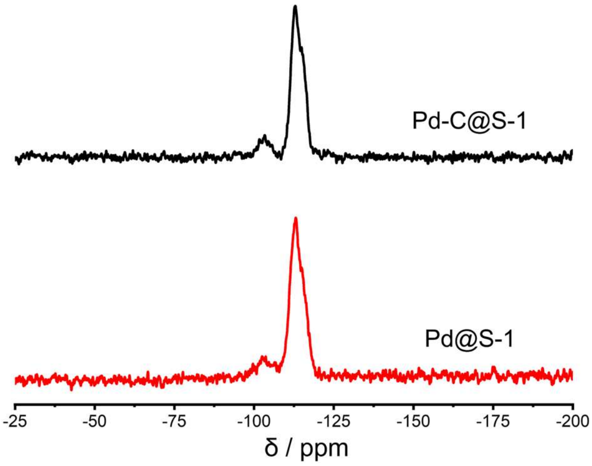

Figure S14. ^{29}Si MAS NMR spectra of Pd-encapsulated samples.

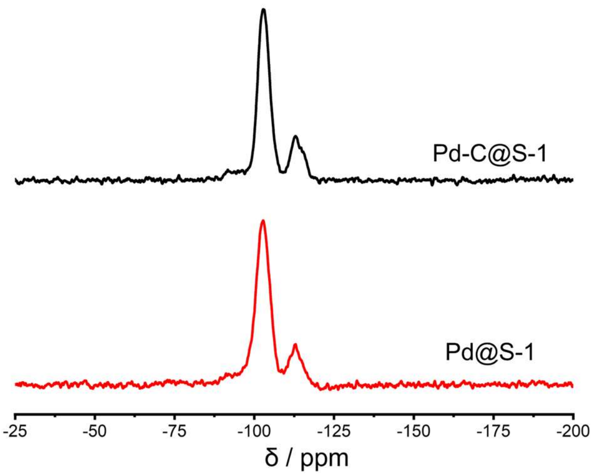

Figure S15. ^{1}H-^{29}Si CP MAS NMR spectra of Pd-encapsulated samples.

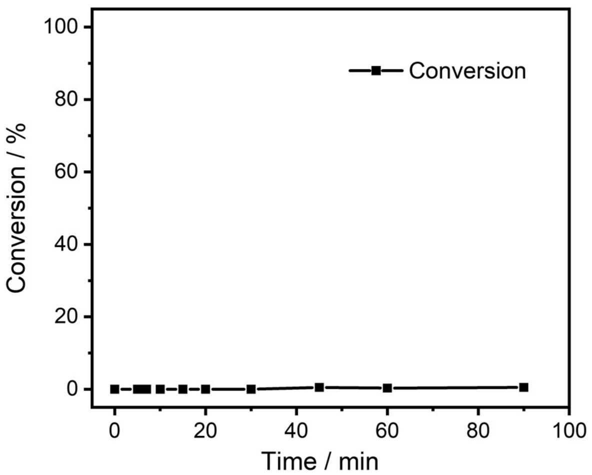

Figure S16. Furfural conversion of C@S-1. Reaction conditions: 0.61 mmol furfural, 30 mg of catalyst, 10 mL H_2O, 5 bar H_2, 80 °C.

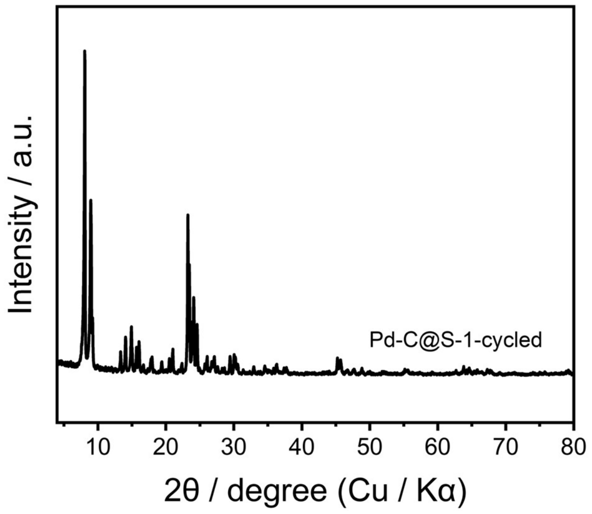

Figure S17. XRD pattern of the recycled Pd-C@S-1.

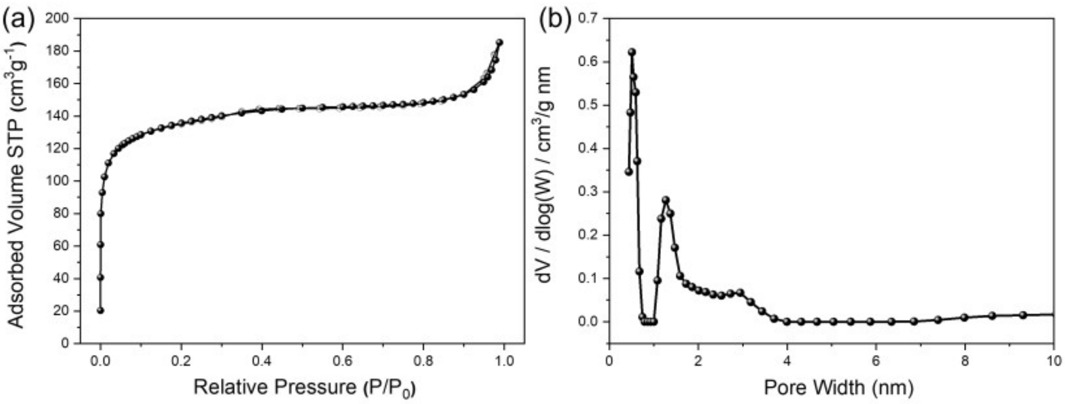

Figure S18. (a) Ar adsorption-desorption isotherm and (b) pore size distribution of the cycled Pd-C@S-1 catalyst.

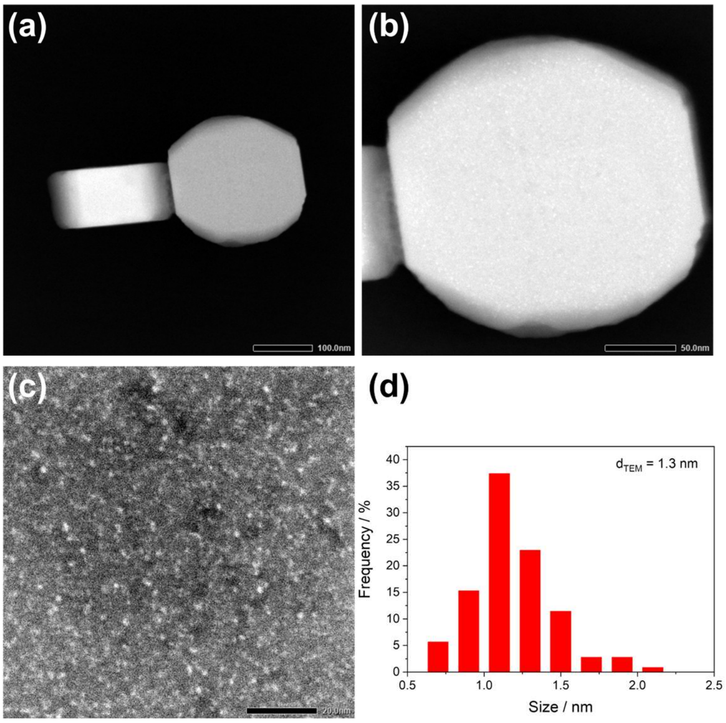

Figure S19. (a-c) TEM images of cycled Pd-C@S-1 and (d) particle size distribution of Pd species.

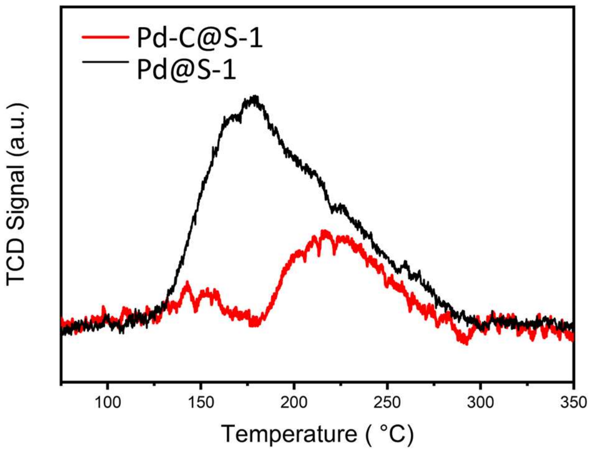

Figure S20. Furfural-TPD over Pd-encapsulated samples.

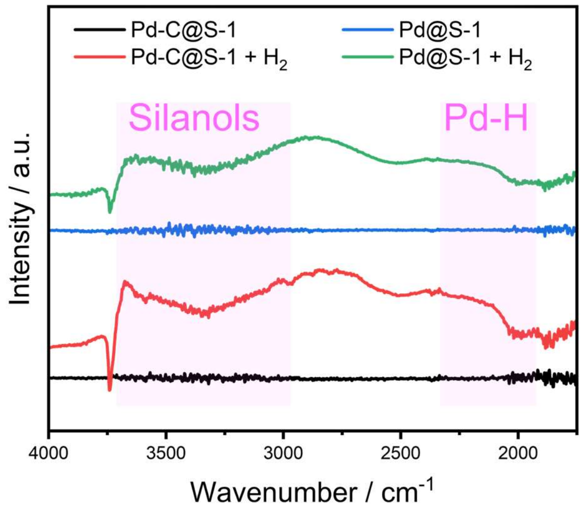

Figure S21. H_2-DRIFT over Pd-encapsulated samples.

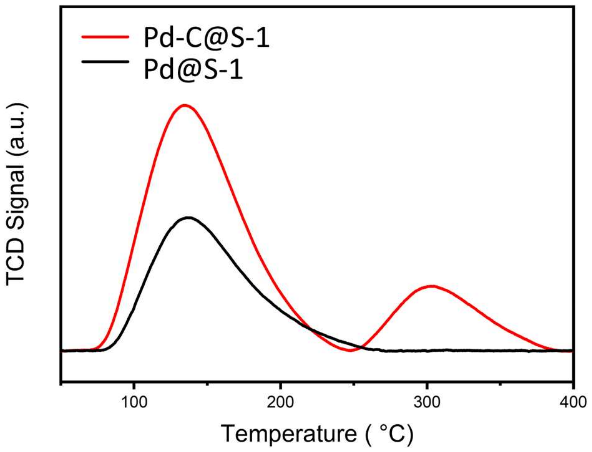

Figure S22. H_2-TPD curves of Pd-encapsulated samples.

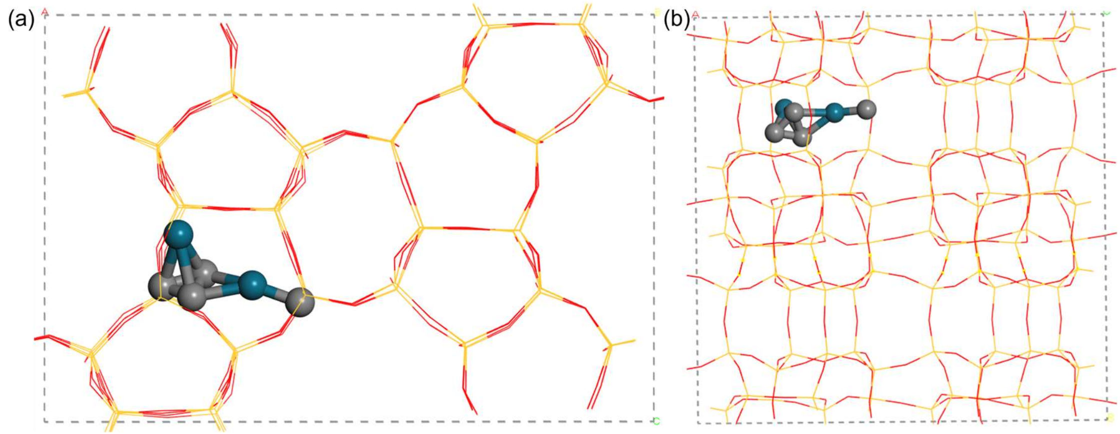

Figure S23. (a) Top and (b) side views of Pd-C@S-1 model.

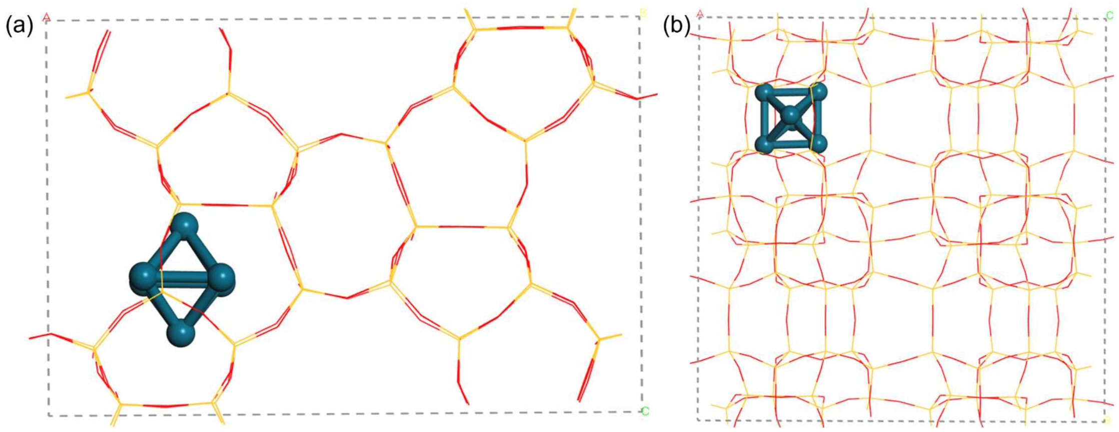

Figure S24. (a) Top and (b) side views of Pd@S-1 model.

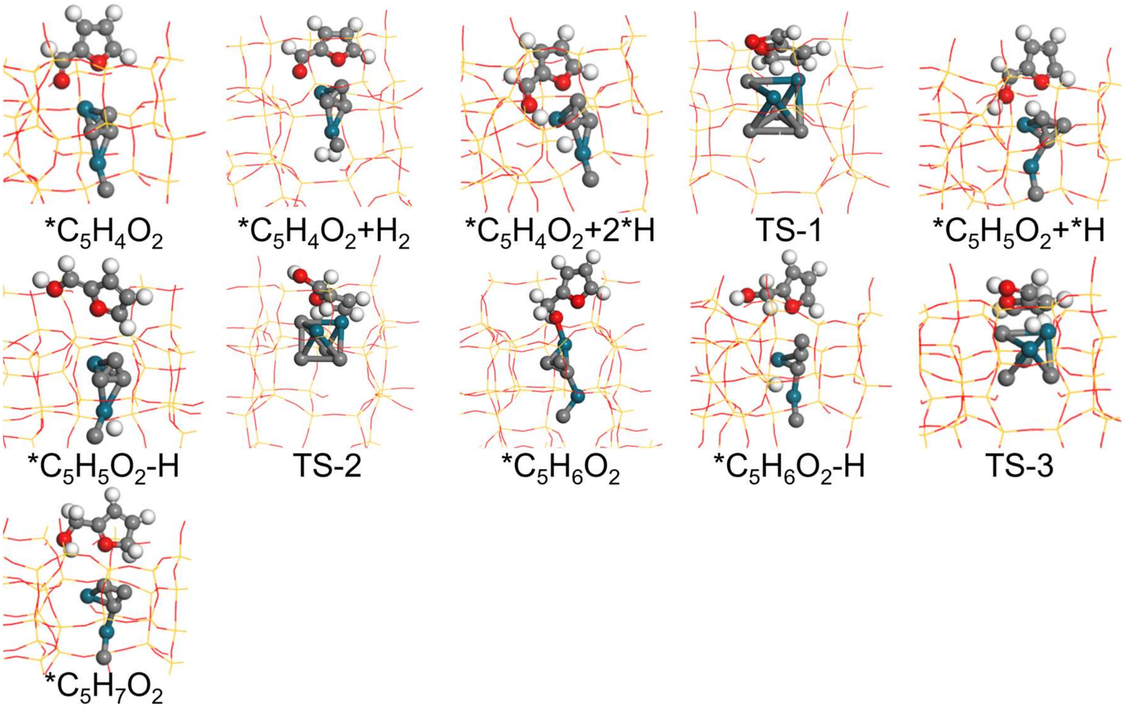

Figure S25. DFT optimized structures of reaction intermediates and transition states in the selective hydrogenation of furfural over Pd-C@S-1.

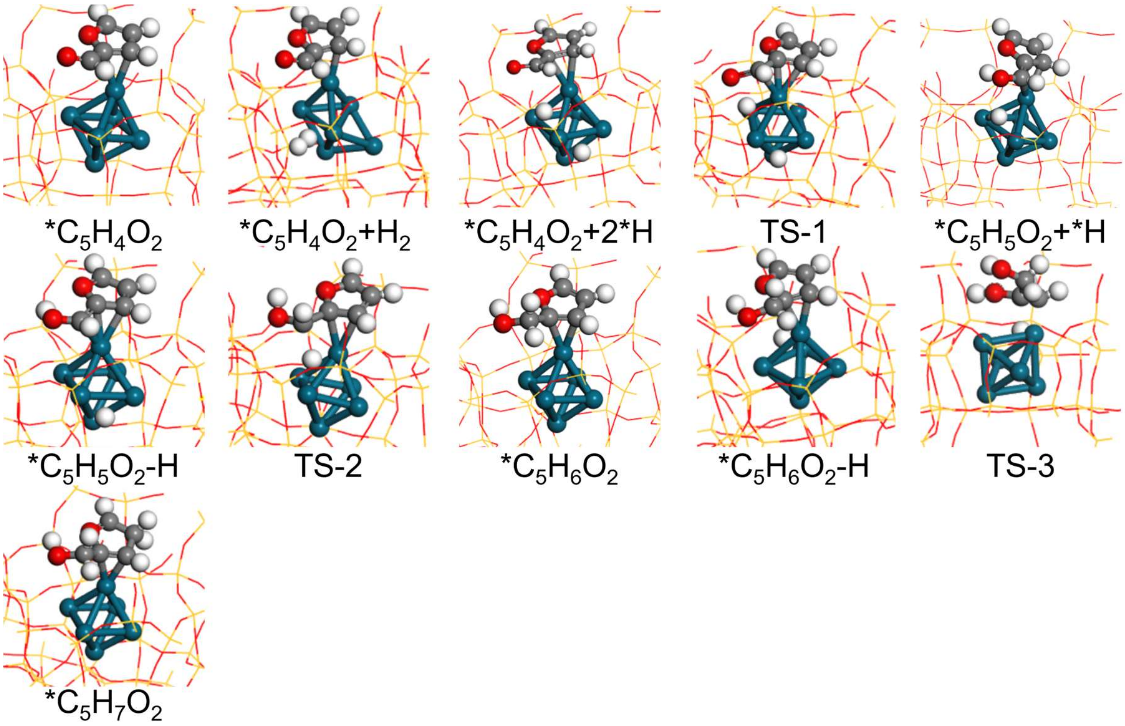

Figure S26. DFT optimized structures of reaction intermediates and transition states in the selective hydrogenation of furfural over Pd@S-1.

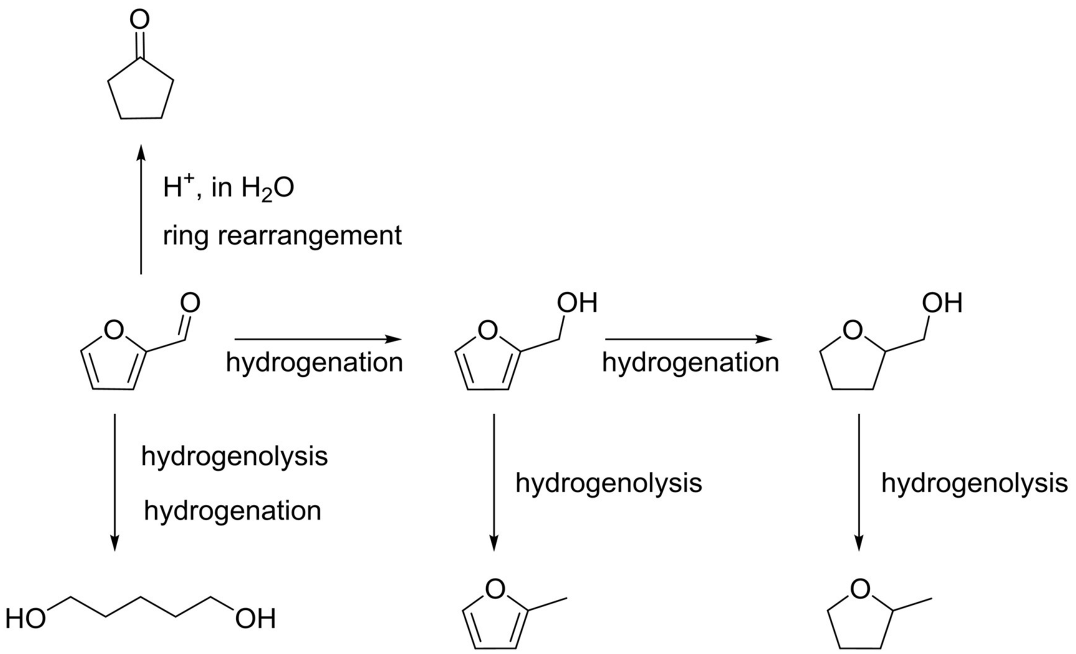

Scheme S1. Reaction Pathways for the Hydrogenation of Furfural.

Table S1. Composition and textural porosities of the samples.

Sample	Pd[a] (wt%)	S_{BET}[b] (m^2/g)	S_{micro}[c] (m^2/g)	S_{meso}[c] (m^2/g)	V_{micro}[c] (cm^3/g)	V_{total}[d] (cm^3/g)
Pd-C@S-1	0.71	464	367	97	0.13	0.23
Pd@S-1	0.72	489	382	107	0.13	0.24
Pd-C@S-1-cycled	0.71	454	351	103	0.13	0.24

[a] Measured by inductively coupled plasma-optical emission spectroscopy (ICP-OES). [b] Specific surface area calculated from argon adsorption isotherm (87 K) using the BET method. [c] S_{micro} (micropore area) and V_{micro} (micropore volume) calculated using the t-plot method, argon adsorption (87 K). [d] Total pore volume at $P/P_0 = 0.99$, argon adsorption (87 K).

Table S2. Structural parameters extracted from the quantitative EXAFS curve-fitting.[a]

Sample	Shell	C.N.[b]	R (Å)[c]	σ^2(Å^2)[d]	ΔE_0 (eV)[e]	R-factor (%)[f]
Pd-C@S-1	Pd-C	3.1±0.9	2.00±0.01	0.0031±0.0020	-2.79±1.59	1.9
	Pd-Pd	2.4±0.5	2.73±0.01	0.0068±0.0017		
Pd@S-1	Pd-O	2.1±0.4	1.99±0.02	0.0067±0.0008	-6.2±0.62	1.6
	Pd-Pd	5.4±0.8	2.73±0.01	0.0067±0.0008		

[a] The value of the amplitude reduction factor (S_0^2 = 0.81) lines between 0.8 and 0.9; [b] C.N. is the coordination number; [c] R is bond distance; [d] σ^2 is Debye-Waller factor (a measure of thermal and static disorder in absorber-scatterer distances); [e] ΔE_0 is edge energy shift (the difference between the zero-kinetic energy value of the sample and that of the theoretical model). [f] R factor is used to value the goodness of the fitting.

Table S3. Compositional analyses of the prepared samples[a]

	C (wt%)	H (wt%)	N (wt%)
Pd-C@S-1	0.54	0.17	0.00
Pden@S-1	9.42	1.85	0.51

[a] Elemental analysis was recorded on a C, H, N elemental analyzer.

References

1. M. Hohwy; H.J. Jakobsen; M. Edén; M.H. Levitt; N.C. Nielsen, *J. Chem. Phys.* 1998, **108**, 2686-2694.

2. G. Qi; Q. Wang; J. Xu; F. Deng, *Chem. Soc. Rev.* 2021, **50**, 8382-8399.

3. F. Brunet; P. Bertani; T. Charpentier; A. Nonat; J. Virlet, *J. Phys. Chem. B* 2004, **108**, 15494-15502.

4. J. Raya; J. Hirschinger; S. Ovarlez; F. Giulieri; A.-M. Chaze; F. Delamare, *Phys. Chem. Chem. Phys.* 2010, **12**, 14508-14514.
